# An Internet-Based Intervention to Promote Mental Fitness for Mildly Depressed Adults: Randomized Controlled Trial

**DOI:** 10.2196/jmir.2603

**Published:** 2013-09-16

**Authors:** Linda Bolier, Merel Haverman, Jeannet Kramer, Gerben J Westerhof, Heleen Riper, Jan A Walburg, Brigitte Boon, Ernst Bohlmeijer

**Affiliations:** ^1^Trimbos Institute (Netherlands Institute of Mental Health and Addiction)Department of Public Mental HealthUtrechtNetherlands; ^2^University of TwenteDepartment of Psychology, Health and TechnologyEnschedeNetherlands; ^3^Trimbos Institute (Netherlands Institute of Mental Health and Addiction)Innovation Centre of Mental Health & Technology I.COMUtrechtNetherlands; ^4^VU UniversityEMGO institute for Health and Care researchAmsterdamNetherlands; ^5^Leuphana UniversityInnovation IncubatorLueneburgGermany; ^6^Trimbos Institute (Netherlands Institute of Mental Health and Addiction)Board of ManagementUtrechtNetherlands

**Keywords:** public health, prevention, depression, well-being, randomized controlled trial, Internet

## Abstract

**Background:**

Depression is a worldwide problem warranting global solutions to tackle it. Enhancing well-being has benefits in its own right and could be a good strategy for preventing depression. Providing well-being interventions via the Internet may have synergetic effects.

**Objective:**

Psyfit (“mental fitness online”) is a fully automated self-help intervention to improve well-being based on positive psychology. This study examines the clinical effects of this intervention.

**Methods:**

We conducted a 2-armed randomized controlled trial that compared the effects of access to Psyfit for 2 months (n=143) to a waiting-list control condition (n=141). Mild to moderately depressed adults in the general population seeking self-help were recruited. Primary outcome was well-being measured by Mental Health Continuum-Short Form (MHC-SF) and WHO Well-being Index (WHO-5); secondary outcomes were depressive symptoms, anxiety, vitality, and general health measured by Center for Epidemiological Studies Depression Scale (CES-D), Hospital Anxiety and Depression Scale Anxiety subscale (HADS-A), and Medical Outcomes Study-Short Form (MOS-SF) vitality and general health subscales, respectively. Online measurements were taken at baseline, 2 months, and 6 months after baseline.

**Results:**

The dropout rate was 37.8% in the Psyfit group and 22.7% in the control group. At 2-month follow-up, Psyfit tended to be more effective in enhancing well-being (nonsignificantly for MHC-SF: Cohen’s *d*=0.27, *P*=.06; significantly for WHO-5: Cohen’s *d*=0.31, *P*=.01), compared to the waiting-list control group. For the secondary outcomes, small but significant effects were found for general health (Cohen’s *d*=0.14, *P*=.01), vitality (*d*=0.22, *P*=.02), anxiety symptoms (Cohen’s *d*=0.32, *P*=.001), and depressive symptoms (Cohen’s *d*=0.36, *P*=.02). At 6-month follow-up, there were no significant effects on well-being (MHC-SF: Cohen’s *d*=0.01, *P*=.90; WHO-5: Cohen’s *d*=0.26, *P*=.11), whereas depressive symptoms (Cohen’s *d*=0.35, *P*=.02) and anxiety symptoms (Cohen’s *d*=0.35, *P*=.001) were still significantly reduced compared to the control group. There was no clear dose–response relationship between adherence and effectiveness, although some significant differences appeared across most outcomes in favor of those completing at least 1 lesson in the intervention.

**Conclusions:**

This study shows that an online well-being intervention can effectively enhance well-being (at least in the short-term and for 1 well-being measure) and can help to reduce anxiety and depression symptoms. Further research should focus on increasing adherence and motivation, reaching and serving lower-educated people, and widening the target group to include people with different levels of depressive symptoms.

**Trial Registration:**

Netherlands Trial Register (NTR) number: NTR2126; http://www.trialregister.nl/trialreg/admin/rctview.asp?TC=2126 (archived by WebCite at http://www.webcitation.org/6IIiVrLcO).

## Introduction

### Relevance of Well-Being

Depression is a highly prevalent problem worldwide [1], which underscores the global urgency of tackling this mental illness [2]. The average lifetime prevalence estimates of major depressive episodes is 15%, with 1 in 20 people suffering from major depressive episodes at any given time [1]. In addition, many more people present subclinical depressive symptoms, putting them at greater risk of going on to develop a full-blown mental disorder [3]. The enhancement of well-being may be just as important in dealing with poor mental health as treating the symptoms of depression [4]. Ample studies show that the level of well-being is a predictor of psychopathology, independent of the influence of negative affect [5,6]. The presence of well-being is also beneficial in its own right. Well-being is associated with a healthier and longer life [7,8], prosocial behavior, and maintenance of high-quality relationships with family and friends [7]. Thus, well-being may play a role both in supporting health and human functioning and in reducing symptoms of mental illness.

Three different types of well-being have been identified in various studies. *Subjective well-being* refers to positive affect and/or life satisfaction [9], *psychological well-being* refers to the level of positive functioning, containing constructs such as meaning in life, goal setting, and mastery [10], and *social well-being* contains constructs such as the level of social integration and social contribution [11].

### Positive Psychology

In the emerging field of positive psychology, interventions aimed at flourishing and positive functioning are being developed and evaluated. These interventions focus on individual competencies and strengths, rather than on mental symptoms and disorders [12]. Examples of interventions are “counting your blessings” [13], “practicing kindness” [14], “expressing gratitude” [13,15], and “using your personal strengths” [13]. Positive psychology also embraces other like-minded traditions, such as mindfulness [16] and life review [17].

Evidence of the effectiveness of positive psychological interventions was reviewed in 2 meta-analyses showing that these interventions enhance well-being and reduce depressive symptoms [18,19]. In addition, positive psychological interventions may reach people who are otherwise more difficult to connect with. People presenting subclinical depression or other problems, such as stress and anxiety, may find a well-being approach more appealing than a problem-oriented approach [20]. One of the main obstacles worldwide in mental health care is to reach people in need of treatment or prevention with good quality interventions [21]. By their inherent appeal, positive psychological interventions may help to narrow this mental health gap.

### Combining Positive Psychological Interventions and the Internet

Providing positive psychological interventions via the Internet may offer synergetic opportunities. The effectiveness of online problem-based interventions (eg, in reducing depressive symptoms or anxiety [22] or problematic alcohol use [23]) has been demonstrated meta-analytically. Internet interventions, especially self-help interventions, may be more affordable and accessible for many people as opposed to face-to-face interventions [24]. Therefore, online positive psychological interventions may offer the most suitable and effective strategy for reaching large target groups. To date, a literature review of the effectiveness of online positive psychological interventions has shown mixed results [25]. In this review, 5 randomized controlled trials were included [26-30], of which 3 demonstrated enhanced well-being [27,29,30] and 3 showed significant symptom reduction [28-30]. The authors concluded that there is still an “effectiveness gap between offline and online formats” in positive psychological interventions [25]. In more recent studies of online positive psychological interventions, mixed results were also found. In a comprehensive study by Gander et al [31], 9 online positive psychological interventions were compared to a placebo exercise. Significant improvement in psychological well-being was found for 8 exercises, but not at all time points, and 5 of 9 exercises reduced depressive symptoms [31]. In the study of Layous et al [32] the online variant of the “best possible selves” exercise was just as effective as the face-to-face variant, and more effective than the placebo intervention. Schueller and Parks [33] showed that it might not always be effective to provide more exercises and content because a 2-exercise and a 4-exercise condition were more effective than the 6-exercise condition.

One of the reasons for a gap between face-to-face and online interventions could be the low adherence rates [25,34]. Adherence may be related to effectiveness such that the more people adhere to an intervention, the larger the effect sizes are [35,36]. This supposed relationship needs further clarification in online positive psychological interventions [25].

### Current Study

The primary goal of the current study was to examine the effectiveness of Psyfit, an online positive psychological intervention, in comparison with a waiting-list control group. This study distinguishes itself from former experimental research, which was limited to single-component interventions [27,29,30] or longer multiple and fixed-sequential interventions [26,28,33], in focusing on a multicomponent, flexible, online intervention. The intervention resembles a toolbox where people can pick and mix their personal training program. From self-determination theory, it can be reasoned that this idea would promote autonomy in the participant, leading to more intrinsic motivation to follow the program [37]. Indeed, experimental studies show that most people are better off with a tailored positive psychological intervention shaped to their personal preferences and needs [38,39].

We hypothesized that the online positive psychological intervention group would demonstrate a significant increase in well-being and a reduction in depressive and anxiety symptoms at 2- and 6-month follow-ups as compared to the control group. The second goal of the study was to examine the role of adherence. We hypothesized that the more people adhered to the intervention, the larger the effects would be. A third goal was to examine whether particular subgroups benefit more or less than others from the intervention (less vs more depressive symptoms at baseline, higher- vs lower-educated people, men vs women, older vs younger people). Because of the broad nature of the intervention, it was hypothesized that each of the subgroups was served equally well.

## Methods

### Study Design

We conducted a randomized controlled trial (NTR2126) of the online positive psychological intervention Psyfit as compared to a waiting-list control group. Online measurements were measured at baseline, 2 months, and 6 months after starting the intervention. Details of the study design and the intervention are reported more extensively in the study protocol that was published previously [40], but a summary is presented subsequently. The study was approved by the Dutch Medical Ethics Committee for Mental Health Care (registration number 9218).

### Intervention

Psyfit is an online self-help intervention, without support from a therapist. Psyfit aims to enhance well-being by stimulating personal growth and positive functioning. A parallel goal is to reduce depressive and anxiety symptoms [41]. The intervention is based on positive psychological principles and addresses strengths and personal competencies rather than mental problems and deficiencies. It incorporates evidence-based exercises based on positive psychology and elements stemming from mindfulness, cognitive behavioral therapy, and problem-solving therapy [42]. In Psyfits’ communication message, an analogy is made with physical fitness. Similar to the saying “an apple a day keeps the doctor away,” Psyfit encourages people to complete their daily mental fitness training.

There are 6 modules in Psyfit, each containing a 4-lesson program: (1) personal mission statement and setting your goals, (2) positive emotions, (3) positive relations, (4) mindfulness, (5) optimistic thinking, and (6) mastering your life. Each week, the lesson consisted of psycho-education and a practical exercise. At the end of the week, participants received an email notifying them that the next lesson could be started. Participants could start or finish modules as they wished, as long as they were in sequence.

Participants allocated to the intervention group received an email with a personal username and password. After logging in, 2-month access to Psyfit was activated. Participants were advised to complete at least 1 module during the intervention period. Each module is a separate module on its own and may, in theory, improve well-being.

### Control Group

Participants in the control group were told they were on a waiting list for 6 months before they received their login codes for Psyfit. A waiting-list control group is ethically acceptable when there is no immediate risk or treatment indication [43], thus fitting the preventive nature of this study. Participants in the control group had unrestricted access to professional help, if needed.

### Participants

**Figure 1:**
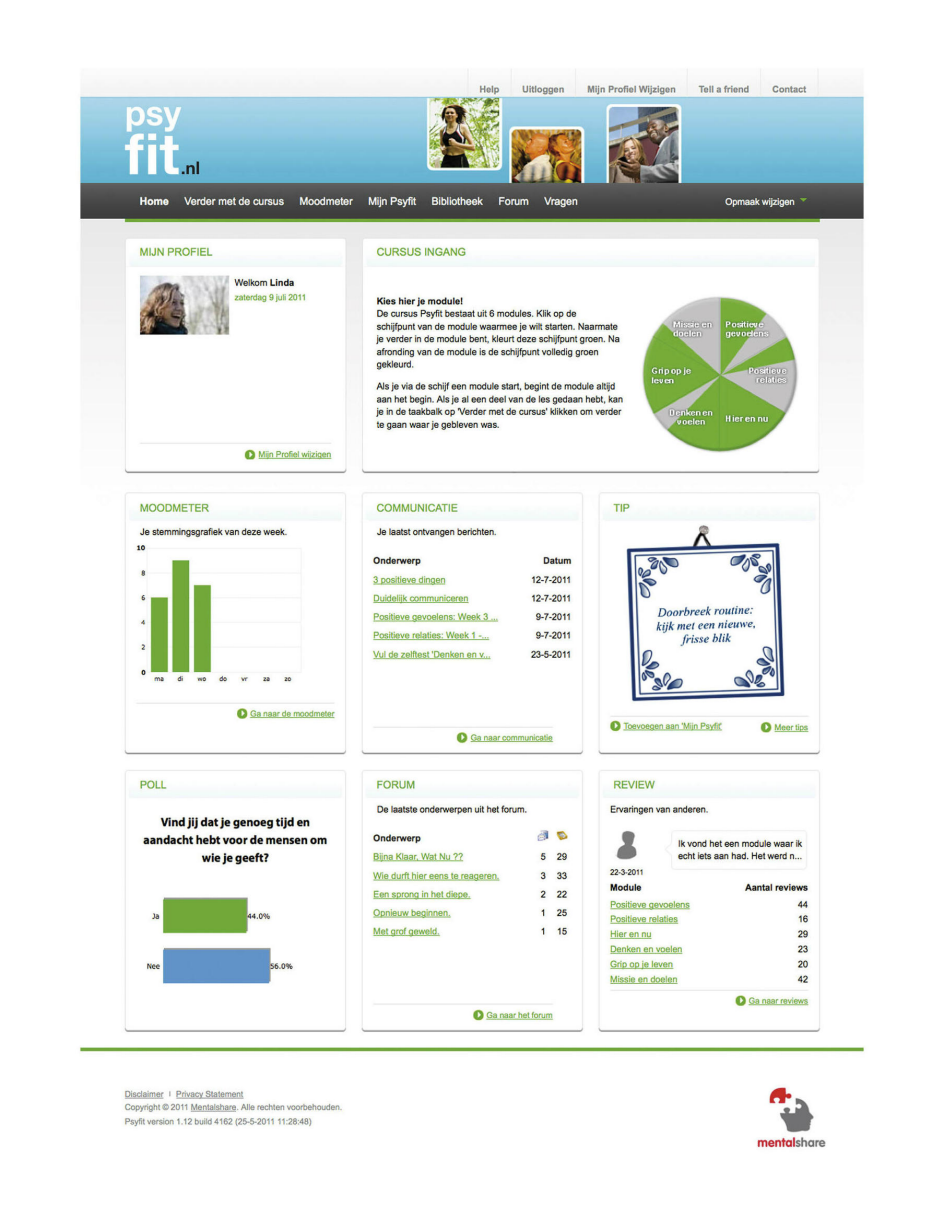
Screenshot of the Psyfit website.

Participants were recruited from the adult well-being-seeking population in the Netherlands in March and April 2010. The recruitment message was formulated positively (not with a focus on symptoms and problems): “Would you like to increase your mental fitness? Improve your mental fitness and participate in our study of an online self-help program.” Banners and advertisements were placed in free newspapers and on Facebook. Interested people registered at the Psyfit website (Figure 1) [44] and subsequently received an email with information on the study and a link to the online consent form and baseline questionnaire. Adult participants (21 years and older) were included when informed consent was obtained and if they presented mild to moderate depressive symptoms as represented by a score of 10-24 on the Center for Epidemiologic Studies Depression Scale (CES-D) [45] and a languishing or moderate level of well-being as measured with the Mental Health Continuum-Short Form (MHC-SF) in which the levels languishing, moderate, and flourishing can be distinguished [46]. Furthermore, they had to have access to a computer and Internet and have sufficient knowledge of the Dutch language. Participants with serious depressive symptoms (CES-D score ≥25) or active suicidal thoughts or plans (question from the Web Screening Questionnaire [47]) were excluded. Those meeting any of these exclusion criteria were advised to seek professional help. Those scoring too high on well-being according to the inclusion criteria were excluded, but were invited to do Psyfit after the study. Participants in both the experimental and control group had unlimited access to professional help, such as primary care and psychological support.

### Randomization

Randomization took place after baseline measurement and was carried out by using a computer-generated randomization list in blocks of 2, stratified by gender, education (higher/lower), and depression symptom level (CES-D scores 10-15 and 16-24).

### Outcomes and Instruments

The primary outcome measure was well-being. This was assessed with 2 questionnaires: the MHC-SF which measures positive mental health in terms of subjective, psychological, and social well-being [46], and the World Health Organization 5-item Well-Being Index (WHO-5) [48,49], a short measure of overall well-being. Secondary outcomes were depressive symptoms, as measured with the CES-D [45,50]; anxiety symptoms, as measured with the Hospital Anxiety and Depression Scale, Anxiety subscale (HADS-A) [51]; and general health and vitality, as measured with 2 subscales from the Medical Outcomes Study 36-Item Short Form (MOS SF-36) [52]. All measurement instruments were self-reported and administered via email with a link to the questionnaire on the Internet. The instruments have been shown to have satisfactory reliability and validity (also see the protocol article for elaboration) [40]. Participant satisfaction was measured using the 8-item Client Satisfaction Questionnaire short form (CSQ-8) [53].

During the trial, usage data from the Web application containing log files were gathered, which enabled monitoring of intervention adherence.

### Analyses

Analyses were conducted following the intention-to-treat (ITT) principle. Accordingly, all participants were analyzed in the group to which they were allocated. Missing data at 2-month and 6-month follow-ups were imputed using the estimation maximization (EM) method in SPSS version 19 (IBM Corp, Armonk, NY, USA). Reporting of the results follows the guidelines of the Consolidated Standards of Reporting Trials (CONSORT) eHealth checklist [54]. We applied unpaired *t* tests and logistic regression analyses to examine possible baseline differences between dropouts and nondropouts. A chi-square test (χ^2^) was used to determine possible differential loss to follow-up between experimental group and control group. Dropout was defined as completing the baseline and 2-month follow-up questionnaire, but not the 6-month follow-up questionnaire, or completing the baseline questionnaire, but neither of the follow-up questionnaires.

The effectiveness of Psyfit was examined by regression analyses. We used the clinical outcomes on continuous measures (MHC-SF, WHO-5, CES-D, HADS-A, and subscales of the MOS SF-36) as dependent variables for the 2- and 6-month follow-ups separately. The intervention dummy and the baseline measurements of the corresponding outcome variables were used as independent variables.

The size of the effect was estimated by using Cohen’s *d*. Cohen’s *d* is calculated as the difference between 2 means divided by the pooled standard deviation. A Cohen’s *d* of 0.5 indicates that the mean of the intervention group is half a standard deviation larger than the mean of the control group. Cohen’s *d* from 0.56 to 1.2 is a large effect size, 0.33 to 0.55 is moderate, and 0 to 0.32 is considered small [55]. We calculated effect sizes (baseline to 2- and 6-month follow-ups, respectively) of each condition separately, and after that calculated the difference between experimental group and control group (∆*d*). As a sensitivity analysis, a completers-only analysis was conducted as well.

To examine the role of adherence, a dose–response relationship was analyzed. Five separate groups were made up of participants adhering to 0, 1, 2, 3, or 4 lessons from any module. Differences between these groups and a possible linear relation (time×group interaction) were investigated by means of a repeated measures ANOVA analysis. The levels of adherence were used as independent variables and the clinical outcomes at 2- and 6-month follow-ups as repeated measures. An unpaired *t* test was used to examine the difference between hardly adhered (adhered to 0 or 1 lesson) and at least some adherence (adhered to 2-4 lessons).

Finally, a moderator analysis was conducted to determine whether certain subgroups (men vs women; education level higher vs lower; mild vs moderate depressive symptoms: CES-D score ≤16 or >16; younger/older age group: ≤45 years or >45 years) benefited more or less from the intervention. This was done by regressing the outcome variable on the independent group variables, the condition dummy, and the interaction between subgroup and condition dummy while controlling for the baseline measurement.

For all analyses, we used 2-sided tests with a significance level of *P*<.05. We only used *P*<.10 for the adherence analysis because of the reduced number of participants and lower power.

## Results

### Flow of Participants

**Figure 2:**
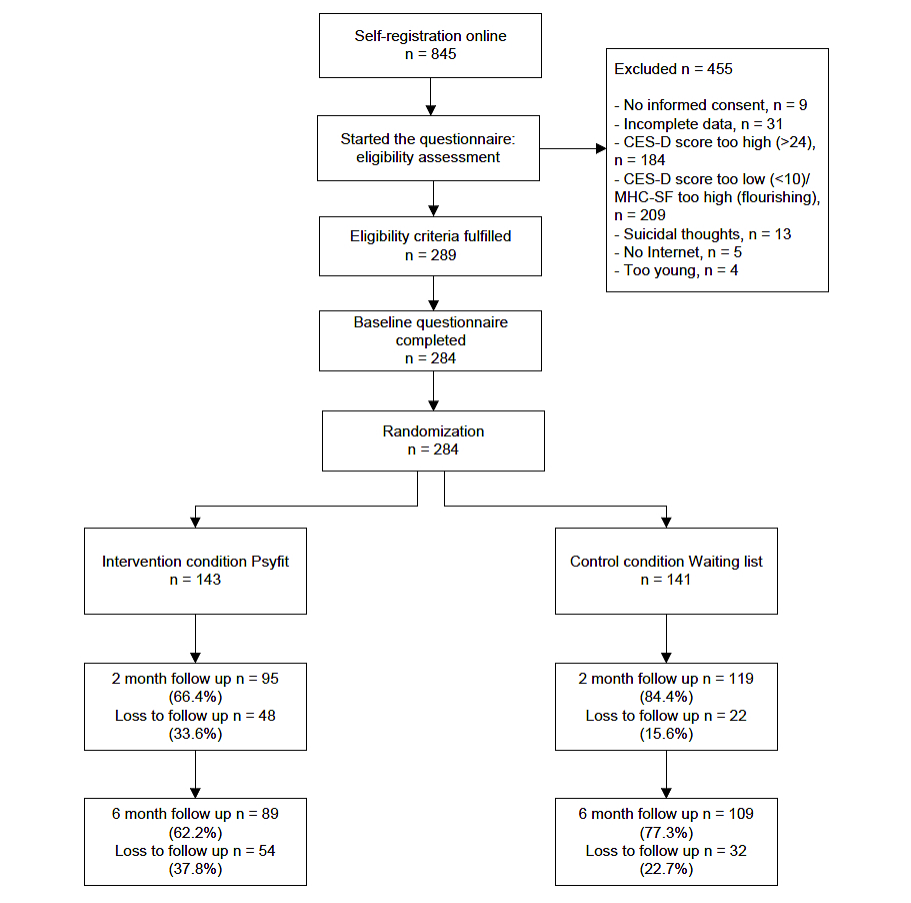
Participants’ flow through the study.

Figure 2 shows the flow of participants. A total of 845 persons were interested in participating in the study and registered with the website. After giving informed consent and filling out the online screening form and baseline questionnaire, 284 people were included in the study (33.6%). Participants were randomly assigned to the intervention (n=143) or the waiting-list control group (n=141).

### Participant Characteristics

The demographic characteristics and baseline scores of the participants are shown in Table 1. The mean age of participants was 43.2 years (SD 11.8) and most were female (226/284, 79.6%). Participants were mostly highly educated (208/284, 73.2%) and most had paid employment (214/284, 75.4%). The mean CES-D depression score at baseline was slightly above the cut-off score of 16 (mean 16.80, SD 4.13) indicating a clinically relevant level of depressive symptoms [50], and the MHC-SF score was below the Dutch national mean of 3.98 (mean 3.47, SD 0.63) [46].

**Table 1:** Baseline characteristics of participants.

Characteristic		Psyfit (n=143)	Control group (n=141)	All (N=284)
Age (years), mean, (SD)		43.5 (11.7)	42.8 (11.9)	43.2 (11.8)
**Age categories, n (%)**				
	21-34 years	36 (25.2)	37 (26.2)	73 (25.7)
	35-64 years	100 (69.9)	101 (71.6)	201 (70.8)
	65-100 years	7 (4.9)	3 (2.1)	10 (3.5)
Female, n (%)		114 (79.7)	112 (79.4)	226 (79.6)
Major negative life event (yes), n (%)		80 (55.9)	73 (51.8)	153 (53.9)
**Education, n (%)**				
	Lower (up to high school and middle-level applied education)	39 (27.3)	37 (26.2)	76 (26.8)
	Higher (academic/professional)	104 (72.7)	104 (73.8)	208 (73.2)
**Daily activities, n (%)**				
	Paid employment	106 (74.1)	108 (76.6)	214 (75.4)
	Unemployed/unable to work	20 (14.0)	10 (7.1)	30 (10.6)
	Other (student, at home, retired)	17 (11.9)	23 (16.3)	40 (14.1)
**Living situation, n (%)**				
	With partner, with or without children	92 (64.3)	84 (59.6)	176 (62.0)
	No partner, with or without children	45 (31.5)	48 (34.0)	93 (32.7)
	Other (dorm or with parents)	6 (4.2)	9 (6.4)	15 (5.3)
**Test scores, mean (SD)**				
	MHC-SF (well-being)	3.45 (0.62)	3.50 (0.63)	3.47 (0.63)
	WHO-5 (well-being)	10.81 (4.31)	11.52 (4.38)	11.17 (4.35)
	CES-D (depression)	16.91 (4.16)	16.67 (4.11)	16.80 (4.13)
	HADS-A (anxiety)	6.55 (2.86)	6.53 (2.96)	6.54 (2.91)
	MOS SF subscale general health	18.57 (3.51)	17.95 (3.61)	18.26 (3.57)
	MOS SF subscale vitality	14.46 (3.16)	14.62 (2.83)	14.54 (3.00)

### Attrition

The response rate was 75.4% (214/284) at 2-month follow-up and 69.7% (198/284) at 6-month follow-up. The response rate was significantly higher in the control group compared to the Psyfit group at 2-month follow-up (84.4% vs 66.4%; χ^2^_1_ = 12.3, *P*<.001) and 6-month follow-up (77.3% vs 62.2%; χ^2^_1_ = 7.6, *P*=.01).

Tested at *P*<.05, dropout analysis revealed that there were several significant differences between dropouts and completers. Those who dropped out were more likely to be living in a dorm or with their parents (χ^2^_2_ = 9.9, *P*=.002) and be of younger age (χ^2^_1_ = 4.2, *P*=.04). No significant differences emerged between dropouts and completers regarding baseline symptoms. Examination of the interaction showed that dropouts in the control group scored significantly lower than dropouts in the Psyfit group on the MOS SF-36 general health subscale, indicating poorer health for dropouts in the control group (*F*_1,280_ = 6.48, *P*=.01).

### Effects of the Intervention

**Figure 3:**
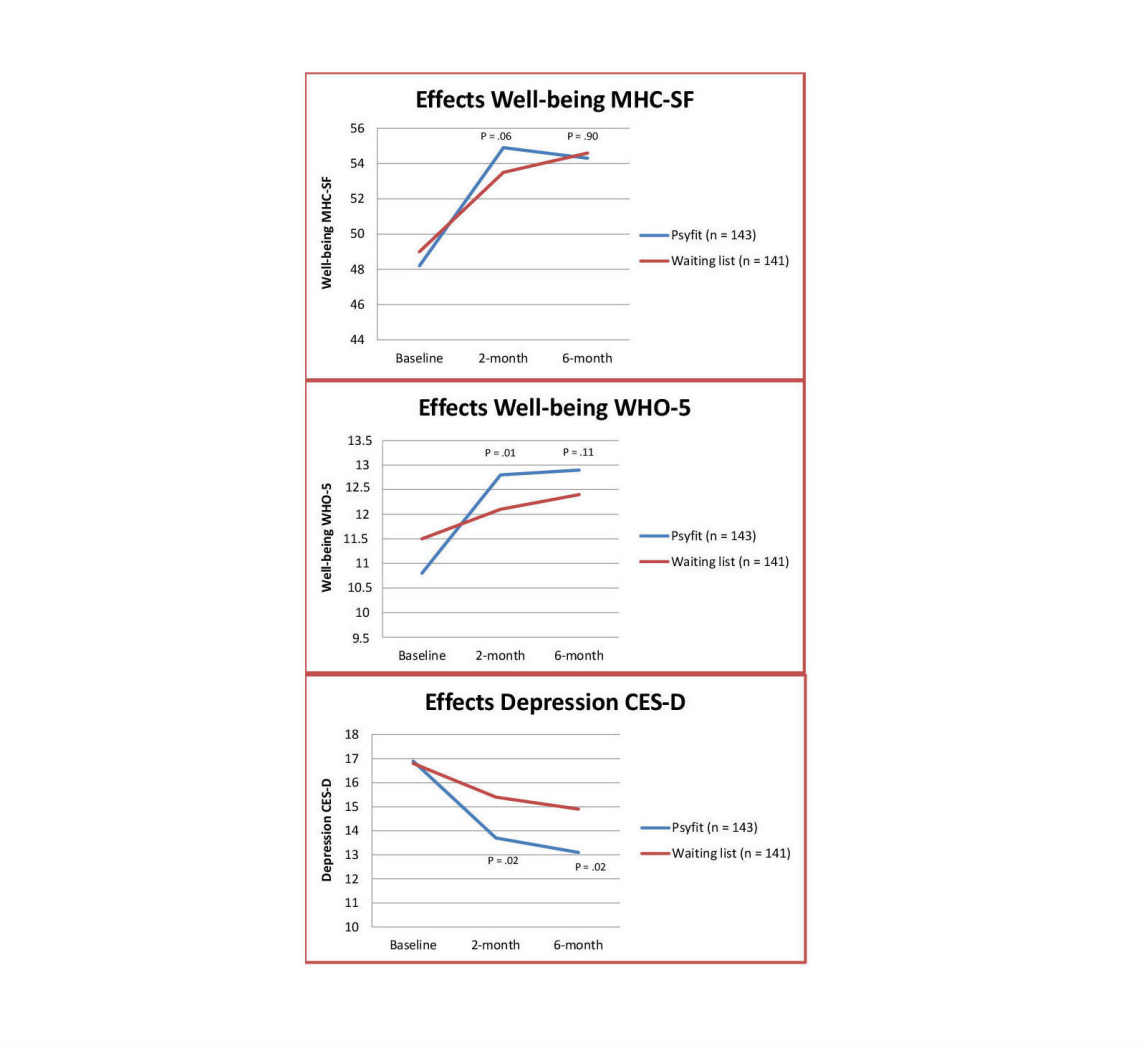
Depicted scores at baseline, 2-, and 6-month follow-ups for the MHC-SF, WHO-5, and CES-D (intention-to-treat sample).

Table 2 presents the means and standard deviations for the outcome measures at 2- and 6-month follow-ups plus effect sizes and the results of the regression analysis in the EM imputed ITT sample. In Figure 3, the results of Psyfit on the MHC-SF, WHO-5, and CES-D are depicted. From baseline to the 2-month follow-up, well-being (the main outcome measure) as measured with the MHC-SF was higher although this did not meet statistical significance (beta = 0.09, *P*=.06). A significant effect on the other well-being measure, the WHO-5, was found (beta = 0.12, *P*=.01). Between-group effects fell within the small range (MHC-SF: *d*=0.27; WHO-5: *d*=0.31). With regard to the secondary outcome measures, the intervention group showed a significant decline at 2-month follow-up in both depressive symptoms (beta = –0.13, *P*=.02) and anxiety (beta = –0.15, *P*=.001), and a significant improvement in both self-reported health (beta = 0.09, *P*=.01) and vitality (beta = 0.12, *P*=.02) versus the control group. Effect sizes were small (MOS SF general health: *d*=0.14; MOS SF vitality: *d*=0.22; HADS-A: *d*=0.32) to medium (CES-D: *d*=0.36). At 6-month follow-up, results were sustained for anxiety (beta = –0.17, *P*=.001) and depressive symptoms (beta = –0.13, *P*=.02), presenting medium effect sizes (both *d*=0.35), but were no longer significant for well-being, health, and vitality.

The same trends in effects emerged in the completers-only analysis: positive effects for all outcomes at 2-month follow-up and sustained effects for anxiety and depressive symptoms at 6-month follow-up (Table 3). However, the effect of well-being at 2-month follow-up as measured with the MHC-SF was not significant in the completers-only analysis (beta = 0.08, *P*=.14, *d*=0.19) nor were there significant effects for depressive symptoms at 6-month follow-up (beta = –0.10, *P*=.14, *d*=0.26). Vitality was improved at 2-month follow-up, but this did not meet statistical significance (beta = 0.11, *P*=.06, *d*=0.19).

**Table 2:** Effects of Psyfit, intention-to-treat analysis.

Measures		Psyfit (n=143)			Control group (n=141)			Linear regression analysis			
		Mean	SD	*d*	Mean	SD	*d*	Beta	*t*_281_	*P* value	∆*d*
**MHC-SF**											
	Baseline	3.45	0.62		3.50	0.63					
	2-month	3.92	0.71	0.71	3.82	0.82	0.44	0.09	1.88	.06	0.27
	6-month	3.88	0.79	0.61	3.90	0.71	0.60	0.01	0.13	.90	0.01
**WHO-5**											
	Baseline	10.81	4.31		11.52	4.38					
	2-month	12.75	4.47	0.44	12.12	4.63	0.13	0.12	2.47	.01	0.31
	6-month	12.92	4.77	0.47	12.45	4.61	0.21	0.09	1.61	.11	0.26
**CES-D**											
	Baseline	16.91	4.16		16.67	4.12					
	2-month	13.67	6.69	0.57	15.39	7.64	0.21	–0.13	–2.33	.02	0.36
	6-month	13.06	7.55	0.63	14.94	7.48	0.28	–0.13	–2.38	.02	0.35
**HADS-A**											
	Baseline	6.55	2.86		6.53	2.96					
	2-month	5.75	2.90	0.28	6.64	3.08	–0.04	–0.15	–3.29	.001	0.32
	6-month	5.65	3.39	0.29	6.70	2.98	–0.06	–0.17	–3.38	.001	0.35
**MOS SF health**											
	Baseline	18.57	3.51		17.95	3.61					
	2-month	19.21	3.16	0.19	18.11	3.50	0.05	0.09	2.72	.01	0.14
	6-month	18.65	3.41	0.02	18.20	3.51	0.07	0.00	–0.68	.95	–0.05
**MOS SF vitality**											
	Baseline	14.46	3.16		14.62	2.83					
	2-month	15.74	3.09	0.41	15.14	2.76	0.19	0.12	2.33	.02	0.22
	6-month	15.70	3.57	0.37	15.20	3.01	0.20	0.09	1.70	.09	0.17

**Table 3:** Effects of Psyfit, completers-only analysis.

Measures		Psyfit^a^			Control group^b^			Linear regression analysis			
		Mean	SD	*d*	Mean	SD	*d*	Beta	*t* (df)	*P* value	∆*d*
**MHC-SF**											
	Baseline	3.52	0.63		3.51	0.63					
	2-month	3.95	0.81	0.59	3.81	0.85	0.40	0.08	1.50 (211)	.14	0.19
	Baseline	3.51	0.61		3.50	0.64					
	6-month	3.89	0.84	0.52	3.91	0.76	0.58	–0.20	–0.32 (195)	.75	–0.06
**WHO-5**											
	Baseline	11.27	4.45		11.45	4.32					
	2-month	12.98	5.11	0.36	11.97	4.78	0.11	0.11	1.98 (211)	.049	0.25
	Baseline	11.07	4.37		11.58	4.45					
	6-month	12.72	5.44	0.33	12.59	4.84	0.22	0.04	0.57 (195)	.57	0.11
**CES-D**											
	Baseline	16.57	4.12		16.55	4.18					
	2-month	13.35	7.97	0.51	15.58	8.11	0.16	–0.14	–2.12 (211)	.04	0.35
	Baseline	17.04	4.14		16.66	4.00					
	6-month	13.35	8.64	0.54	14.87	8.04	0.28	–0.10	–1.50 (195)	.14	0.26
**HADS-A**											
	Baseline	6.27	2.83		6.52	3.03					
	2-month	5.49	3.18	0.26	6.68	3.24	–0.05	–0.16	–2.89 (211)	.004	0.31
	Baseline	6.28	2.78		6.57	2.95					
	6-month	5.55	3.78	0.22	6.69	3.23	–0.04	–0.14	–2.24 (195)	.03	0.26
**MOS SF health**											
	Baseline	18.66	3.50		17.97	3.66					
	2-month	19.40	3.31	0.22	18.09	3.61	0.03	0.11	2.59 (211)	.01	0.19
	Baseline	18.39	3.40		18.39	3.35					
	6-month	18.57	3.58	0.05	18.61	3.36	0.07	–0.01	–0.11 (195)	.91	–0.02
**MOS SF vitality**											
	Baseline	14.67	3.17		14.66	2.82					
	2-month	15.80	3.39	0.34	15.09	2.86	0.15	0.11	1.88 (211)	.06	0.19
	Baseline	14.37	3.29		14.80	2.83					
	6-month	15.53	3.99	0.32	15.39	3.15	0.20	0.048	0.74 (195)	.46	0.12

^a^2-month follow-up: n=95, 6-month follow-up: n=89.

^b^2-month follow-up: n=119, 6-month follow-up: n=109.

### Adherence

On average, 1.39 modules were started (SD 1.45). Most participants (74/143, 51.7%) started 1 module (mode and median). Participants most often enrolled in the positive emotions module (51/143, 35.7%), before personal mission statement and setting your goals (44/143, 30.8%), mindfulness (31/143, 21.7%), and optimistic thinking (29/143, 20.3%). Positive relations (22/143, 15.4%) and mastering your life (22/143, 15.4%) were the least popular modules.

In Table 4, different adherence grades and their effect sizes are shown for the experimental group (ITT sample). Adherence was defined as completing zero (31/143, 21.7%), 1 (46/143, 32.6%), 2 (24/143, 16.8%), 3 (29/143, 20.3%), or 4 lessons (13/143, 9.1%) from 1 or more modules. In plots, there is no clear dose–response relationship (the more adherence, the larger the effect size) recognizable. Tested at *P*<.10, repeated measures analysis (Table 5) showed significant effects for the MHC-SF at both 2- and 6-month follow-ups after completing 2 lessons (*F*_8,274_ = 1.72, *P*=.09, Cohen’s *d* pre-post 1.25 and 1.18). Significant effects were found for the MOS-SF general health subscale at 2-month follow-up after completing 4 lessons (*F*_8,274_ = 2.16, *P*=.03, Cohen’s *d* pre-post 0.71) and for the MOS-SF vitality subscale at 2-month follow-up after adhering to 2 lessons (*F*_8,274_ = 1.75, *P*=.09, Cohen’s *d* pre-post 0.74). When examining hardly any adherence (completing ≤1 lesson) versus at least some adherence (completing ≥2 lessons), 2 comparisons appeared to be significant (not presented in Tables 4 and 5). The effect sizes of the HADS-A anxiety scale at 2-month follow-up (*d*=0.16 vs *d*=0.43, *t*_141_ = 2.24, *P*=.03) and of the CES-D depression scale at 6-month follow-up (*d*=0.47 vs *d*=0.87, *t*_141_ = 1.84, *P*=.07) were significantly higher for the at least some adherence group compared to those who completed 1 lesson or less.

### Participant Satisfaction

Results regarding participant satisfaction are based on the completers sample (93/95 filled in the satisfaction questionnaire). Regarding participant satisfaction, 40 of 93 participants (43.0%) in the experimental group expressed satisfaction with Psyfit. The other 57.0% (53/93) were indifferent and/or dissatisfied. In the questionnaire, we were unable to separate the indifferent and the dissatisfied categories. Psyfit was found to be somewhat helpful in dealing with problems by 47.3% of the participants (44/93). Almost 70% (65/93) of the participants would recommend Psyfit to friends, and 66.7% (62/93) would do Psyfit (again), if needed. Adherence was positively related to satisfaction rate (χ^2^_3_ = 15.0, *P*=.02).

### Subgroup Effects

A moderator analysis with well-being (MHC-SF and WHO-5) and depression (CES-D) as dependent variables was conducted to explore which subgroups benefited more or less from the intervention. The only significant result was found for education level on the WHO-5 well-being measure at 6-month follow-up (*d*=0.46 for higher-educated participants vs *d* = –0.20 for lower-educated participants; *F*_1,280_ = 6.23, *P*=.01). Other nonsignificant trends for educational level were found on WHO-5 well-being at 2-month follow-up (*d*=0.42 vs *d*=0.02; *F*_1,280_ = 3.60, *P*=.06) and on the CES-D at 6-month follow-up (*d*=0.58 vs *d* = –0.13, *F*_1,280_ = 3.66, *P*=.06), suggesting that higher-educated people profited more from the intervention than lower-educated people on these measures. For age, a nonsignificant trend was found on the CES-D at 6-month follow-up (*d*=0.61 for participants older than 45 years vs *d*=0.09 for younger participants, *F*_1,280_ *= 3.62, P* = .06), suggesting that the older group benefited more from Psyfit. For the other 2 potential moderators, gender and depression status, no significant or relevant interaction effects were found.

**Table 4:** Adherence grades and effect sizes, intention-to-treat sample (experimental group, Cohen’s *d* pre-post).

Measures		<1 lesson (n=31)		1 lesson (n=46)		2 lessons (n=24)		3 lessons (n=29)		≥4 lessons (n=13)	
		Mean (SD)	*d*	Mean (SD)	*d*	Mean (SD)	*d*	Mean (SD)	*d*	Mean (SD)	*d*
**MHC-SF**											
	Baseline	3.46 (0.60)		3.46 (0.70)		3.17 (0.58)		3.52 (0.52)		3.69 (0.62)	
	2-month	3.84 (0.61)	0.63	3.90 (0.64)	0.66	4.05 (0.81)	1.25	3.92 (0.68)	0.66	3.99 (1.04)	0.36
	6-month	3.74 (0.69)	0.43	3.87 (0.79)	0.55	3.98 (0.78)	1.18	3.94 (0.76)	0.65	3.91 (1.09)	0.25
**WHO-5**											
	Baseline	10.00 (3.59)		11.74 (4.22)		9.75 (5.33)		10.41 (4.45)		12.31 (3.22)	
	2-month	12.00 (3.21)	0.59	12.83 (4.01)	0.26	13.34 (6.24)	0.62	12.09 (4.01)	0.40	14.69 (5.60)	0.52
	6-month	11.56 (3.91)	0.42	13.28 (4.76)	0.34	13.29 (5.48)	0.65	12.80 (4.93)	0.51	14.55 (4.86)	0.54
**CES-D**											
	Baseline	17.71 (4.19)		16.28 (3.71)		17.58 (4.74)		17.41 (4.33)		14.92 (3.73)	
	2-month	15.21 (6.21)	0.47	13.73 (5.77)	0.53	13.99 (9.74)	0.47	13.61 (6.77)	0.67	9.31 (5.92)	1.13
	6-month	14.06 (6.51)	0.67	14.42 (7.67)	0.31	12.16 (8.56)	0.78	12.03 (7.94)	0.84	9.85 (5.90)	1.03
**HADS-A**											
	Baseline	6.90 (2.53)		6.52 (2.96)		7.04 (3.21)		6.00 (3.11)		6.15 (2.03)	
	2-month	6.28 (2.47)	0.25	6.22 (2.88)	0.10	5.58 (3.62)	0.35	4.71 (2.58)	0.45	5.00 (2.80)	0.47
	6-month	6.16 (3.19)	0.26	6.01 (3.37)	0.16	6.08 (4.00)	0.26	4.62 (2.94)	0.46	4.61 (3.40)	0.55
**MOS SF health**											
	Baseline	18.52 (2.74)		18.46 (3.82)		19.00 (3.95)		19.14 (3.55)		17.00 (3.06)	
	2-month	18.98 (2.74)	0.17	19.05 (3.14)	0.17	19.47 (4.05)	0.12	19.48 (2.80)	0.11	19.31 (3.43)	0.71
	6-month	17.82 (2.81)	–0.25	19.07 (3.29)	0.17	18.88 (4.41)	–0.03	19.06 (3.30)	0.02	17.85 (3.34)	0.27
**MOS SF vitality**											
	Baseline	14.03 (2.77)		15.04 (3.17)		13.96 (3.97)		14.00 (2.87)		15.38 (2.84)	
	2-month	15.20 (2.16)	0.48	15.83 (3.29)	0.24	16.83 (3.81)	0.74	15.21 (2.82)	0.43	15.85 (3.24)	0.16
	6-month	15.03 (2.77)	0.36	15.90 (3.64)	0.25	15.85 (4.23)	0.46	16.01 (3.67)	0.61	15.62 (3.91)	0.07

**Table 5:** Repeated measures analysis on adherence grades (see Table 4).

Measures	Time		Time×group	
	*F*_2,137_	*P*	*F*_8,274_	*P*
MHC-SF	34.20	<.001	1.72	.09
WHO-5	18.06	<.001	0.95	.48
CES-D	30.00	<.001	0.99	.44
HADS-A	9.20	<.001	0.53	.83
MOS SF health	14.29	<.001	2.16	.03
MOS SF vitality	13.53	<.001	1.75	.09

## Discussion

### Principal Results

This randomized controlled trial examined the efficacy of an online self-help course aimed at promoting mental fitness and subsequent well-being. The results at 2-month follow-up show that intervention group participants made a significant improvement in the level of overall well-being on one measure (WHO-5) than participants in the waiting-list control group and a nonsignificant improvement on the other well-being measure (MHC-SF). Furthermore, general health and vitality level were significantly enhanced, and depression and anxiety symptoms were reduced in comparison to the waiting-list control group. At 6-month follow-up, effects were maintained for depression and anxiety. All effects were in the small to medium range. Adherence analysis revealed no clear dose–response effect, although some larger effects appeared for people receiving at least a minimal part of the intervention. For well-being (only WHO-5) and depression, somewhat larger effects were found for higher-educated participants.

### Comparison With Previous Work

The effects of taking part in Psyfit are comparable with effects of similar positive psychological interventions in self-help format with regard to well-being (0.14-0.33 in a meta-analysis at immediate follow-up), but appear to be larger for depression on average (0.15 in the same meta-analysis) [19]. When compared with separate studies of other online positive self-help interventions, the effect sizes at immediate follow-up in the current study are quite similar, and higher in some cases. For example, in the Seligman et al study [29], exercises that worked well had effect sizes of 0.23 for well-being and 0.14-0.28 for depression. In the study of Shapira and Mongrain [30], effect sizes of 0.03-0.18 were found for well-being and 0.09-0.30 for depression. Mitchell et al [27] found effect sizes ranging from 0.05 to 0.29 for well-being and an effect size of -0.17 for depression. At longer-term follow-up, effect sizes of the current study were comparable or higher for depression (up to 0.33 for Seligman [29], 0.42 for Shapira [30], and -0.04 for Mitchell [27]), and lower for well-being (up to 0.28 for Seligman [29], up to 0.30 for Shapira [30], and up to 0.33 for Mitchell [27]).

### Adherence and Acceptability

More than three-quarters (78.3%) of the participants in the intervention group completed at least 1 lesson in Psyfit. The personal mission statement and setting your goals module and the positive emotions module were the most popular modules, each chosen by one-third of the total sample. This can be considered a satisfactory adherence rate in general because it is comparable with, or higher than, adherence rates in other online self-help interventions [34]. However, from a practical point of view, it is questionable whether this adherence rate deserves our endorsement because less than 10% fully adhered to 1 module (consisting of 4 lessons). The group who completed at least 1 lesson showed slightly larger effect sizes than the whole group analysis, indicating that following the self-help course could predict effect size to some extent, but these results were not convincing. The explanation for this is not immediately clear. Dose-response effects (the more adherence, the larger the effects) have been established in several online trials of mental health promotion [35]. On the other hand, not every trial demonstrated such an adherence effect [56], which is comparable to our results. Unfortunately, we collected no information on the reasons for quitting (or not even starting) the intervention. One possible explanation is that nonparticipants/dropouts felt disappointed, or not capable of proceeding with the intervention. We did determine that adherence rate was positively related to satisfaction rate. Alternatively, it could be argued that people stopped because they felt better and were no longer in need of help, which could explain the modest effects of adherence, implying that the nonadhering participants were doing better as well.

Still, the question of how to increase the engagement and adherence for this type of intervention is important. It is likely that by increasing adherence, people could benefit more. The incorporation of persuasive and gaming elements into an intervention may hold considerable promise because such elements may raise client satisfaction and can encourage participants to stick to the intervention [36,57,58]. Less than half (approximately 40%) of participants were satisfied with Psyfit, although a larger percentage (approximately 65%) would recommend the program to a friend or would do it again if needed. Persuasive elements may help to arouse and prolong participant motivation, which may ultimately contribute to sustainable behavioral change [57]. Although Psyfit already contains some engaging and motivating elements, such as email reminding and self-monitoring instruments, other persuasive functionalities could be added to make it a truly interactive and personalized device, such as tailored feedback and action planning [57]. In addition, minimal guidance (eg, telephone/email contact or coaching supervision) is worth considering because this may increase adherence and effectiveness. There is some experimental evidence showing that tailoring positive psychological exercises to needs and preferences can indeed effectively enhance intervention engagement and adherence [39], although preference for a certain well-being activity appears to be not enough to guarantee intervention effectiveness [41]. For this reason, it seems warranted to ensure a good person-activity fit; a kind of diagnosis to determine which well-being enhancing exercise suits the participant best [38]. Features of the intervention (dosage and variety) as well as personal aspects, such as motivation and efficacy beliefs, should be taken into account [59]. Lastly, it has been found that lower-educated groups often lack the more sophisticated Internet skills that are needed to participate in an online intervention [60]. Increasing those skills in these groups might contribute to improved adherence and better performance in online interventions such as Psyfit.

### Population Approach

A relatively slight increase in the overall level of health in a sizeable part of the population may have a larger preventive effect than targeting only the much smaller group of people who are already ill [61,62] . This principle may apply even more for mental health and well-being because stigma surrounding the formal use of mental health services may deter people in need from seeking help [63]. Considering that many people can be reached by Psyfit in a nonstigmatized way and in a relatively short length of time, even the small effect sizes that were demonstrated in this trial may contribute to an important improvement in population mental health. On the other hand, this trial only included a specific target group of people presenting mild to moderate depressive symptoms, which affects generalizability to the larger population. In a naturalistic study, it has been found that people seeking self-help interventions to improve well-being either show rather severe depression scores or hardly any signs of depression [41], whereas the present study targeted people with mild depressive symptoms. It would be insightful to examine if Psyfit is also effective in these other groups in a way that could be generalized to a larger population.

### Limitations

A number of limitations in this study have to be recognized. First, there was a rather high attrition rate and differential dropout between the intervention arms. Although not an uncommon phenomenon in online trials [64], the dropout may have affected the results in a way that is not easy to predict. Thus, the results of our trial should be considered with caution. Second, the intervention used in this study was designed like a toolbox from which people could pick and mix their own personalized program. This can be considered a strength because it enables participants to tailor their own program, which is a unique feature of the intervention. However, it may also be considered a weakness because no reliable statements can be given about the comparative effectiveness of modules and other effective elements of the intervention. Third, we did not measure motivational level, self-efficacy, or readiness to change as is constructed, for example, in the Stages of Change theory by Prochaska and DiClemente [65]. Therefore, we could not examine whether the more motivated and better-equipped people adhered better to the intervention and accordingly benefited more. Fourth, the study used a waiting-list control group. This means there was no blinding of participants and possible placebo effects could not be established. Fifth, the CSQ-8 [53] contains ambiguous reply categories. As a result, some of the satisfaction rates were difficult to interpret. For further research, we recommend that these categories be adapted to an unambiguous Likert scale, for example. Sixth, the effect sizes that were found were mostly in the small range. The study was powered to detect medium-sized effects [40] and may, therefore, be underpowered to detect these small changes (eg, the nonsignificant finding for well-being/MHC-SF at 2-month follow-up).

### Conclusion and Recommendations for Practice and Research

To the best of our knowledge, this is the first study of an online positive psychological intervention with a flexible multicomponent format to demonstrate both small, significant effects on well-being (for one of the measures) and on symptoms of mental disorder. Regarding the implications for public health, Psyfit could be used as an open and highly accessible mental health promotion tool, disseminated by relevant lifestyle and health-related websites and Internet forums, or referred to by health care professionals.

Regarding the future research agenda for online positive psychological interventions, emphasis should be placed on (1) increasing adherence and motivation by using persuasive design and/or providing minimal support, (2) determining what works best for whom and ensure a good person–activity fit, (3) serving lower-educated people better, and (4) considering the use of other control groups to overcome the limitations of a waiting-list control group. The target group could be expanded to present more variety in the depressive symptom spectrum. This will likely help to strengthen the generalizability of these results to a larger group of people.

One of the strengths of this study was the uncomplicated recruitment of participants (845 interested people within a 5-week timeframe), whereas in other randomized controlled trials of online health interventions recruitment can be a serious challenge [66]. As such, Psyfit and other online positive psychological interventions can be regarded as positive technology, a recently proposed concept that refers to the use of technology for improving the quality of personal experience by fostering positive emotions, self-empowerment, and social connectedness [67]. These technologies have the potential to evolve further and become available to many more people. As nonstigmatizing and nonmedicalized tools to address the promotion of mental health and subsequent prevention of depression, they contain the promise of a sustained positive effect on health care and society.

## Multimedia Appendix 1

CONSORT-EHEALTH checklist V1.6.2 [54].

## Abbreviations

CES-D: Center for Epidemiologic Studies Depression Scale
CSQ-8: 8-item Client Satisfaction Questionnaire-short form
EM: estimation maximization
HADS-A: Hospital Anxiety and Depression Scale, Anxiety subscale
ITT: intention-to-treat
MHC-SF: Mental Health Continuum-Short Form
MOS SF-36: Medical Outcomes Study 36-Item Short Form
WHO-5: World Health Organization 5-item Well-Being Index
