# Supplementary material for: An Internet-Based Intervention to Promote Mental Fitness for Mildly Depressed Adults: Randomized Controlled Trial
Source: J Med Internet Res. 2013 Sep 16;15(9):e200. doi: 10.2196/jmir.2603 (PMC3929047; doi:10.2196/jmir.2603)
Supplement: Supplementary file 1 [file jmir_v15i9e200_app1.pdf]

CONSORT-EHEALTH Checklist V1.6.2 Report

(based on CONSORT-EHEALTH V1.6), available at [http://tinyurl.com/consort-ehealth-v1-6].

2603

Date completed

6/13/2013 18:35:56

by

Linda Bolier

Psyfit, Internet-based intervention to promote mental fitness for mildly depressed adults: results of a randomized controlled trial

TITLE

1a-i) Identify the mode of delivery in the title

Yes, we call the mode of delivery 'Internet-based intervention'. E-mail reminding is part of the intervention.

1a-ii) Non-web-based components or important co-interventions in title

No, the e-mail reminding is not mentioned because it is an element in the intervention, though not a central element.

1a-iii) Primary condition or target group in the title

Yes: 'for mildly depressed adults' is in the title.

ABSTRACT

1b-i) Key features/functionalities/components of the intervention and comparator in the METHODS section of the ABSTRACT

Yes, but not in a elaborate way: Psyfit ('mental fitness online') is a fully automated self-help intervention based on positive psychology with the aim of improving well-being.'
The comparator condition is called the 'a waiting list control condition'.

1b-ii) Level of human involvement in the METHODS section of the ABSTRACT

Yes, in the abstract is stated: 'Psyfit ('mental fitness online') is a fully automated self-help intervention...'.

1b-iii) Open vs. closed, web-based (self-assessment) vs. face-to-face assessments in the METHODS section of the ABSTRACT

Yes: 'Online measurements were taken at baseline, 2 months and 6 months after baseline.'

We did not mention the blinding process, because a waiting list control group implies automatically 'unblinded'.

1b-iv) RESULTS section in abstract must contain use data

Number of participants, the primary and secondary outcomes and the relation of adherence to effectiveness are all mentioned in the abstract:

'Drop-out rate was 37.8% in the Psyfit group and 22.7% in the control group. At post-test, Psyfit tended to be more effective in enhancing wellbeing (MHC-SF, Cohen’s d = 0.27, P = .06; WHO-5, Cohen’s d = 0.31, P = .01), compared to the waiting list control group. For the secondary outcomes, small but significant effects were found for general health (Cohen’s d = 0.14, P = .01), vitality (d = 0.22, P = .02), anxiety symptoms (Cohen’s d = 0.32, P = .001), and depressive symptoms (Cohen’s d = 0.36, P = .02). At follow-up, there were no significant effects on the main outcome - well-being - (MHC-SF, Cohen’s d = 0.01, P = .90; WHO-5, Cohen’s d = 0.26, P = .11), whereas depressive symptoms (Cohen’s d = 0.35, P = .02) and anxiety symptoms (Cohen’s d = 0.35, P = .001) were still significantly reduced, as compared to the control group. There was no clear dose-response relationship between adherence and effectiveness, although some significant differences appeared across most outcomes in favour of those completing at least one lesson in the intervention. '

1b-v) CONCLUSIONS/DISCUSSION in abstract for negative trials

No, because there were positive results.
Main conclusion is stated, and recommandations made on the basis of limited uptake and lack of effects for lower educated people.

INTRODUCTION

2a-i) Problem and the type of system/solution

Yes, there is a clear problem analysis:
- First, on the relevance of well-being 'The enhancement of well-being may well be just as important in dealing with poor mental health as is treating the symptoms of depression.' Et cetera
- Second, on the evidence base of positive psychological interventions. 'Evidence of the effectiveness of positive psychological interventions (PPIs) was reviewed in two meta-analyses showing that these interventions enhance well-being and reduce depressive symptoms [18,19]. In addition, PPIs may reach people that are otherwise more difficult to connect with.' Et cetera
- Three, on the potential synergy between positive psychology & Internet-based interventions. 'Internet interventions, especially self-help interventions, may be potentially more affordable and accessible for many people, as opposed to face-to-face interventions [24]. Therefore, online positive psychological interventions (OPPIs) may offer the most suitable and effective strategy for reaching large target groups.' et cetera

After that, there is a statement on the added value of this study: 'This study distinguishes itself from former experimental research, which was limited to single-component interventions [27,29,30] or longer multiple and fixed-sequential interventions [26,28,33], in focussing on a multi-component and flexible online intervention. The intervention resembles a toolbox where people can ‘pick and mix’ their personal training program. From self-determination theory it can be reasoned that this idea would promote autonomy in the participant, leading to more intrinsic motivation to follow the program [37]. Indeed, experimental studies show that most people are better off with a tailored program shaped to their personal preferences and needs [38,39]. '

2a-ii) Scientific background, rationale: What is known about the (type of) system

Yes, there is a short review on the evidence-base of similar interventions, offline and online (see 2a-i).

The choice of the comparator is explained: 'A wait-listed design was chosen in order not to withhold any of the participants from taking part in the intervention.'

METHODS

3a) CONSORT: Description of trial design (such as parallel, factorial) including allocation ratio

Yes, the hypotheses are clearly stated in the paper:

'We hypothesized that the OPPI group would demonstrate a significant increase in well-being and a reduction in depressive and anxiety symptoms at 2-month and 6-month follow-up as compared to the control group. The second goal of the study was to examine the role of adherence. We hypothesized that the more people adhered to the intervention, the larger the effects would be. A third goal was to examine whether particular subgroups benefit more or less than others from the intervention (less versus more depressive symptoms at baseline, higher versus lower educated people, men v. women). Because of the broad nature of the intervention it was hypothesized that each of the subgroups was served equally well.'

3b) CONSORT: Important changes to methods after trial commencement (such as eligibility criteria), with reasons

No, there were no changes to methods.

3b-i) Bug fixes, Downtimes, Content Changes
No, there were no major bug fixes, down times or content changes.
4a) CONSORT: Eligibility criteria for participants
Yes, eligibility criteria were described:
'Adult participants (21 years and older) were included when informed consent was obtained and if they presented mild to moderate depressive symptoms (score 10-24 on the CES-D[44]); and a languishing or moderate level of well-being (as measured with the MHC-SF in which the levels 'languishing', 'moderate' and 'flourishing' can be distinguished [45]). Furthermore, they had to have access to a computer and Internet and have sufficient knowledge of the Dutch language. Participants with serious depressive symptoms (CES-D score >=25) or active suicidal thoughts or plans (question from the Web Screening Questionnaire [46]) were excluded. Those meeting any of these exclusion criteria were advised to seek professional help. Those scoring too high on well-being according to the inclusion criteria were told they could do Psyfit after the study.'
4a-i) Computer / Internet literacy
Computer and Internet literacy was explicitly described:

' Furthermore, they had to have access to a computer and Internet and have sufficient knowledge of the Dutch language.'
4a-ii) Open vs. closed, web-based vs. face-to-face assessments:
Recruitment was online and offline (this was more elaborately done in the protocol article):

'Banners and advertisements were placed in free newspapers and on Facebook.'

The assessments were done online: 'All measurement instruments were self-report and administered via e-mail with a link to the questionnaire on the Internet.'
4a-iii) Information giving during recruitment
This was more extensively done in the protocol article (see also this CONSORT EHEALTH checklist).

But there were some statements in the article:

'The recruitment message was formulated positively (not with a focus on symptoms and problems): "Would you like to increase your mental fitness? Improve your mental fitness and participate in our study of an online self-help program". Banners and advertisements were placed in free newspapers and on Facebook. Interested people registered at the website www.psyfit.nl (Figure 1: [43]) and subsequently received an e-mail with information on the study and a link to the online consent form and baseline questionnaire. '
4b) CONSORT: Settings and locations where the data were collected
Yes, it was an open trial, there was no particular setting.

' Participants were recruited from the adult 'well-being seeking' population in the Netherlands in March and April 2010. '
4b-i) Report if outcomes were (self-)assessed through online questionnaires
Yes: All assessments were done online: 'All measurement instruments were self-report and administered via e-mail with a link to the questionnaire on the Internet.'
4b-ii) Report how institutional affiliations are displayed
No, this is not reported, nor is the impact measured. But it is a good suggestion.
5) CONSORT: Describe the interventions for each group with sufficient details to allow replication, including how and when they were actually administered
5-i) Mention names, credential, affiliations of the developers, sponsors, and owners
Yes, this is mentioned:

'Conflicts of interest
Trimbos Institute is the developer of Psyfit.nl.'

' Acknowledgements
We are grateful to Jolanda Lourens, Jorne Grolleman, Monique Hulsbergen, Katherina Martin Abello and Iris Rosier for their help with the development of the Psyfit intervention. The study is funded by the Dutch Ministry of Health, Welfare and Sport.'

5-ii) Describe the history/development process
This was described in the protocol paper.
5-iii) Revisions and updating
No, this was the first version of Psyfit, and only this version was used for the trial.
5-iv) Quality assurance methods
No, it was not mentioned in the paper, but all important safety and privac standards are applicated in Psyfit.
5-v) Ensure replicability by publishing the source code, and/or providing screenshots/screen-capture video, and/or providing flowcharts of the algorithms used
We have provided a screenshot of the intervention.
5-vi) Digital preservation
Yes, this was captured (reference number 43).
5-vii) Access
Yes, but very shortly (more comprehensively in the protocol article):

'Participants allocated to the intervention group received an email with a personal username and password. After logging in, two-month access to Psyfit was activated. Participants were advised to complete at least one module during the intervention period. '

On request, login demos are available.
5-viii) Mode of delivery, features/functionalities/components of the intervention and comparator, and the theoretical framework
There descriptions of the program in the article, however, for a comprehensive overview we would like to refer to the protocol article and to the accompanying CONSORT EHealth checklist.
5-ix) Describe use parameters
Yes, 'After logging in, two-month access to Psyfit was activated. Participants were advised to complete at least one module during the intervention period. Each module is a separate module on its own and may in theory improve well-being. '

5-x) Clarify the level of human involvement
Yes, 'Psyfit is an online self-help intervention, without support from a therapist. '

In the protocol article is also mentioned that there was a contact form where participants could as questions, both content questions and technical questions.
5-xi) Report any prompts/reminders used
The reminding system was already explained in the protocol paper (and CONSORT checklist).
5-xii) Describe any co-interventions (incl. training/support)
There are no co-interventions.
6a) CONSORT: Completely defined pre-specified primary and secondary outcome measures, including how and when they were assessed
Yes. However, this was more extensively covered in the protocol article.

' Outcomes and instruments
The primary outcome measure was well-being. This was assessed with two scales: the MHC-SF which measures positive mental health in terms of subjective, psychological, and social well-being [45]; and the WHO-5 [47,48], a short measure of overall well-being. Secondary outcomes were depressive symptoms, as measured with the CES-D [44,49]; anxiety symptoms, as measured with the HADS-A [50]; and general health and vitality, as measured with two subscales from the MOS-SF -36 [51]. All measurement instruments were self-report and administered via e-mail with a link to the questionnaire on the Internet. They have been shown to have satisfactory reliability and validity (see also the protocol article for an elaboration on this part) [40]. Participant satisfaction was measured using the Client Satisfaction Questionnaire-short form (CSQ-8) [52].
During the trial, usage data of the web application containing log files were gathered, which enabled monitoring of intervention adherence.'

6a-i) Online questionnaires: describe if they were validated for online use and apply CHERRIES items to describe how the questionnaires were designed/deployed
We would like to refer again to the protocol article and checklist for the answer to this question.
6a-ii) Describe whether and how "use" (including intensity of use/dosage) was defined/measured/monitored
Yes, 'During the trial, usage data of the web application containing log files were gathered, which enabled monitoring of intervention adherence.'
6a-iii) Describe whether, how, and when qualitative feedback from participants was obtained
During the trial, through the contact form we received some feedback from participants.
In the formative stage, focus groups were conducted to gather qualitative information used for the development of the course.
6b) CONSORT: Any changes to trial outcomes after the trial commenced, with reasons
There were no major changes.
The only thing was that in the first week we expanded the age group from 25 to 21 years.
7a) CONSORT: How sample size was determined
7a-i) Describe whether and how expected attrition was taken into account when calculating the sample size
The power calculation was already stated in the protocol article.
7b) CONSORT: When applicable, explanation of any interim analyses and stopping guidelines
There were no interim analyses.
8a) CONSORT: Method used to generate the random allocation sequence
Yes, 'Randomization took place after baseline measurement and was carried out using a computer generated randomization list in blocks of two, stratified by gender, education (high/other) and depression symptom level (CES-D scores 10-15 and 16-24).'
8b) CONSORT: Type of randomisation; details of any restriction (such as blocking and block size)
Yes, '... in blocks of two, stratified by gender, education (high/other) and depression symptom level (CES-D scores 10-15 and 16-24).'
9) CONSORT: Mechanism used to implement the random allocation sequence (such as sequentially numbered containers), describing any steps taken to conceal the sequence until interventions were assigned
The sequence was unknown to the researchers. This was done by a computer program.
10) CONSORT: Who generated the random allocation sequence, who enrolled participants, and who assigned participants to interventions
A computer program generated the allocation sequence. The first en second author assigned the participants.
11a) CONSORT: Blinding - If done, who was blinded after assignment to interventions (for example, participants, care providers, those assessing outcomes) and how
11a-i) Specify who was blinded, and who wasn't
There was no blinding in our study. People assigned to Psyfit knew that they were in the intervention group. There were no care providers.
11a-ii) Discuss e.g., whether participants knew which intervention was the "intervention of interest" and which one was the "comparator"
The participants knew their condition group, see 11a-i.
11b) CONSORT: If relevant, description of the similarity of interventions
Not applicable.
12a) CONSORT: Statistical methods used to compare groups for primary and secondary outcomes
Yes, 'The effectiveness of Psyfit, was examined by regression analyses. We used the clinical outcomes on continuous measures (MHC-SF & subscales, WHO-5, CES-D, HADS-A, subscales of the MOS SF-36) as dependent variables for the 2-month and 6-month follow-up separately. The control group dummy and the baseline measurements of the corresponding outcome variables were used as independent variables.
The size of the effect was estimated by using Cohen's d. Cohen's d is calculated as the difference between two means divided by the pooled standard deviation. A Cohen's d of 0.5 indicates that the mean of the intervention group is half a standard deviation larger than the mean of the control group. Cohen's d from 0.56 to 1.2 can be assumed to be large, 0.33 to 0.55 is moderate, and 0 to 0.32 is considered small [54]. We calculated effect sizes (pre – 2-month, pre – follow-up 6-month) of each condition separately, and after that calculated the difference between experimental group and control group (Δd). As a sensitivity analysis, a completers only-analysis was conducted as well.'
12a-i) Imputation techniques to deal with attrition / missing values
The imputation technique is described:

'Analyses were conducted following the intention-to-treat (ITT) principle. Accordingly, all participants were analysed in the group to which they were allocated. Missing data at 2-month and 6-month follow-up were imputed using the Estimation Maximization (EM) method in the Statistical Package for the Social Sciences (SPSS), version 19.'
12b) CONSORT: Methods for additional analyses, such as subgroup analyses and adjusted analyses

The adherence analysis and moderator analysis is also described:
'To examine the role of adherence, a dose-response relationship was analysed. Five separate groups were made up of participants adhering to 0, 1, 2, 3 or 4 lessons from any module?. Differences between these groups and a possible linear relation (time*group interaction) were investigated by means of a Repeated Measures ANOVA analysis. The levels of adherence were used as independent variables and the clinical outcomes at 2-month and 6-months follow-up as repeated measures.
Finally, a moderator analysis was conducted to determine whether certain groups (men versus women, education level, mild versus moderate depressive symptoms) benefited more or less from the intervention. This was done by regressing the outcome variable on the independent group variables, the condition dummy and the interaction between subgroup and condition dummy, while controlling for the baseline measurement.'

RESULTS

13a) CONSORT: For each group, the numbers of participants who were randomly assigned, received intended treatment, and were analysed for the primary outcome

All these numbers are outlines. e.g.:

'Figure 2 shows the flow of participants. A total of 845 persons were interested in participating in the study and registered with the website. After giving informed consent and filling out the online screening form and baseline questionnaire, 284 people were included in the study (33.6%). Participants were randomly assigned to the intervention (n = 143) or waiting list control group (n = 141).'

And dose received:
'Adherence was defined as completing zero (n = 31, 21.7%), one (n = 46, 32.6%), two (n = 24, 16.8%) three (n = 29, 20.3%), or four lessons (n = 13, 9.1%) from one or more modules.'

13b) CONSORT: For each group, losses and exclusions after randomisation, together with reasons

This was stated in the Attrition analysis. The reasons for drop-out and non-compliance were not explored.

'Attrition
Response rate was 75.4% (n = 214) at 2-month follow-up and 69.7% (n = 198) at 6-month follow-up. The response rate was significantly higher in the control group compared to the Psyfit group at 2-month follow-up (84.4% versus. 66.4%, $\chi^2 = 12.34$, $P < .001$) and 6-month follow-up (77.3% versus. 62.2%, $\chi^2 = 7.63$, $P = .01$).

Tested at $P < .05$, drop-out analysis reveals that there were several significant differences between drop-outs and completers.. Those who dropped out were more likely to be living in a student's home or with their parents ($\chi^2 = 9.93$, $P = .002$) and of younger age ($\chi^2 = 4.15$, $P = .04$). No significant differences emerged between drop-outs and completers regarding baseline measures of the corresponding outcome measures. Examination of the interaction showed that drop-outs in the control group scored significantly lower than drop-outs in the Psyfit group on the MOS SF subscale general health, indicating poorer health for drop-outs in the control group ($F = 6.48$, $P = .01$).'

13b-i) Attrition diagram

Attrition' was in our study 'drop-out', thus not filling in the questionnaires.

The adherence of participants was also extensively covered:

'Adherence was defined as completing zero (n = 31, 21.7%), one (n = 46, 32.6%), two (n = 24, 16.8%) three (n = 29, 20.3%), or four lessons (n = 13, 9.1%) from one or more modules.'

See table 3.

14a) CONSORT: Dates defining the periods of recruitment and follow-up

The recruitment period was stated:
'Participants were recruited from the adult 'well-being seeking' population in the Netherlands in March and April 2010.'

And the periods of the follow up:

'Online measurements were taken at baseline, 2 months, and 6 months after starting the intervention.'

14a-i) Indicate if critical "secular events" fell into the study period

No, there were no critical 'secular events'.

14b) CONSORT: Why the trial ended or was stopped (early)

Not applicable.

15) CONSORT: A table showing baseline demographic and clinical characteristics for each group

This is Table 1 Baseline characteristics.

15-i) Report demographics associated with digital divide issues

We mentioned age, education level, gender, job yes/no and partner yes/no.
Computer/Internet skills were not assessed.

16a) CONSORT: For each group, number of participants (denominator) included in each analysis and whether the analysis was by original assigned groups

16-i) Report multiple "denominators" and provide definitions

In Table 2 Effects of Psyfit, intention-to-treat analysis all N's and effect sizes of the main analysis are reported.
Use of the intervention/Adherence is reported in Table 3.

16-ii) Primary analysis should be intent-to-treat

Primary analysis was intention-to-treat. A sensitivity analyses consisted of a completers analysis.

17a) CONSORT: For each primary and secondary outcome, results for each group, and the estimated effect size and its precision (such as 95% confidence interval)

See Table 2.
And 'Effects of the intervention
Table 2 presents the means and standard deviations for the outcome measures at 2-month and 6-month follow-up plus effect sizes and the results of the regression analysis in the EM imputed intention-to-treat sample. In Figure 3 the results of Psyfit on the MHC-SF, WHO-5 and CES-D are depicted. From baseline to the 2-month follow-up a marginally significant effect was found on well-being - the main outcome measure - as measured with the MHC-SF (Beta = 0.09, P = .06) and a significant effect on the other well-being measure, the WHO-5 (Beta = 0.12, P = .01). Between-group effects fall within the small range (MHC-SF, d = 0.27; WHO-5, d = 0.31). Analysing the subscales of the MHC-SF separately, only the subscale psychological well-being was significant (Beta = 0.15, P = .002, d = 0.39). With regard to the secondary outcome measures, the intervention group showed a significant decline at 2-month follow-up in both depressive symptoms (Beta = -0.13, P = .02) and anxiety (Beta = -0.15, P = .001) and a significant improvement in both self-reported health (Beta = 0.09, P = .01) and vitality (Beta = 0.12, P = .02), versus the control group. Effect sizes are small (MOS SF general health, d = 0.14; MOS SF vitality, d = 0.22; HADS-A, d = 0.32) to medium (CES-D, d = 0.36). At 6 month follow-up, results were sustained for anxiety (Beta = -0.17, P = .00) and depressive symptoms (Beta = -0.13, P = .02), presenting medium effect sizes (both d = 0.35), but were no longer significant at follow-up for well-being, health and vitality.

The same trends in effects emerged in the completers-only analysis (positive effects for all outcomes at 2-month follow-up, sustained effects for anxiety and depressive symptoms at 6-month follow-up). However, the effect of well-being at 2-month follow-up as measured with the MHC-SF is not significant in the completers-only analysis (Beta = 0.08, P = .14, d = 0.19) (while it was significant in the ITT analysis); nor were there significant effects for vitality at 2-month follow-up (Beta = 0.11, P = .06, d = 0.19) or depressive symptoms at 6-month follow-up (Beta = -0.10, P = .14, d = 0.26).'

17a-i) Presentation of process outcomes such as metrics of use and intensity of use

Yes. adherence is defined and a dose-respons analysis is provided.See Table 3 Adherence grades and effect sizes. ITT sample (experimental group, Cohen's d pre- post), Repeated Measures analysis.

17b) CONSORT: For binary outcomes, presentation of both absolute and relative effect sizes is recommended

There were no binary outcomes.

18) CONSORT: Results of any other analyses performed, including subgroup analyses and adjusted analyses, distinguishing pre-specified from exploratory

An adherence analysis and subgroup analysis was also performed and reported:

'Adherence
On average, 1.39 modules were started (Sd = 1.45). Most participants started 1 module (mode and median, n = 74). Participants most often enrolled in the module ‘Positive emotions’ (n = 51, 35.7%), before ‘Personal mission statement and setting your goals’ (n = 44, 30.8%), ‘Mindfulness’ (n = 31, 21.7%) and ‘Optimistic thinking’ (n = 29, 20.3%). ‘Positive relations’ (n = 22, 15.4%) and ‘Mastering your life’ (n = 22, 15.4%) were the least popular modules.
In Table 3 different adherence grades and their effect sizes are shown for the experimental group (ITT sample). Adherence was defined as completing zero (n = 31, 21.7%), one (n = 46, 32.6%), two (n = 24, 16.8%) three (n = 29, 20.3%), or four lessons (n = 13, 9.1%) from one or more modules. In plots there is no clear dose-response relationship (the more adherence, the larger the effect size) recognizable. Tested at P < .10, repeated measures analysis shows significant effects for the MHC-SF at both 2-month and 6-month follow-up after completing two lessons (F = 1.72, P = .09). Significant effects were found for the MOS-SF general health subscale at 2-month follow-up after completing four lessons (F = 2.16, P = .03) and for the MOS-SF vitality subscale at 2-month follow-up after adhering to two lessons (F = 1.75, P = .09). When examining ‘hardly any adherence’ (completing <= 1 lesson) versus ‘at least some adherence’ (completing at least 2 lessons), two comparisons appeared to be significant. The effect sizes of the HADS-A anxiety scale at 2-month follow-up (d = 0.16 versus d = 0.43, t141 = 2.24, P = .03) and of the CES-D depression scale at 6-month follow-up (d = 0.47 versus d = 0.87, t141 = 1.84, P = .07) were significantly higher for the ‘at least some adherence group’, compared to those who completed just one lesson, or less.
Regarding participant satisfaction, 40 out of 93 participants (43%) in the experimental group expressed satisfaction with Psyfit. Almost 70% (65 out of 93) of the participants would recommend Psyfit to friends, and 67% (62 out of 93) would do Psyfit (again) if need be. Adherence is positively related to satisfaction rate (χ^2 = 14.98, df = 3, P = .02).

Subgroup effects
A moderator analysis, with well-being (MHC-SF and WHO-5) and depression (CES-D) as dependent variables, was conducted to explore which subgroups benefited more or less from the intervention. Marginally significant interactions were found only for education level on the WHO-5 well-being measure at 2-month follow-up (d = 0.42 for higher educated participants versus d = 0.02 for lower educated participants, F = 3.60, P = .06) and on the CES-D at 6-month follow-up (d = 0.58 versus d = -0.13, F = 3.66, P = .06). A significant interaction effect on the WHO-5 at 6-month follow-up was likewise found (d = 0.46 versus d = -0.20, F = 6.23, P = .01), indicating that higher educated people profited more from the intervention than lower educated people on these measures. For the other two potential moderators, gender and depression status, no significant interaction effects were found, although there was a recognizable trend that the more depressed people benefited more from the intervention than those with very mild levels of depression.'

18-i) Subgroup analysis of comparing only users

We covered this by saying in the limitations section that we did not measured motivational level and therefore could not interfere if there was a self-selected sample in the group that adhered and fillled in questionnaires.

19) CONSORT: All important harms or unintended effects in each group

We reported the negative effects for people with lower education:

'Marginally significant interactions were found only for education level on the WHO-5 well-being measure at 2-month follow-up (d = 0.42 for higher educated participants versus d = 0.02 for lower educated participants, F = 3.60, P = .06) and on the CES-D at 6-month follow-up (d = 0.58 versus d = -0.13, F = 3.66, P = .06). A significant interaction effect on the WHO-5 at 6-month follow-up was likewise found (d = 0.46 versus d = -0.20, F = 6.23, P = .01), indicating that higher educated people profited more from the intervention than lower educated people on these measures.'

19-i) Include privacy breaches, technical problems

None reported.

19-ii) Include qualitative feedback from participants or observations from staff/researchers

There were no qualitative reports (exept before the intervention was developed).

DISCUSSION

20) CONSORT: Trial limitations, addressing sources of potential bias, imprecision, multiplicity of analyses

20-i) Typical limitations in ehealth trials

The limitations:

'Limitations
A number of limitations in this study have to be recognized. First, there was a rather high attrition rate, and in addition, differential drop-out between the intervention arms. Although not an uncommon phenomenon in online trials [62], the drop-out may have affected the results in a way that is not easy to predict. The results of our trial should thus be considered with caution. Second, the intervention used in this study was designed like a toolbox from which people could pick and mix their own personalized program. This can be considered a strength as it enables participants to tailor their own program, which is a unique feature of the intervention. However, it may also be considered a weakness, as no reliable statements can be given about the effective elements of the intervention. Third, we did not measure motivational level, self-efficacy or 'readiness to change' as is for example constructed in the Stages of Change theory by Prochaska and DiClemente [63]. Therefore we could not examine whether the more motivated and better equipped people adhered better to the intervention and accordingly benefited more. Fourth, the study used a waiting list control group. This means there was no blinding of subjects and possible placebo effects could not be established.'

21) CONSORT: Generalisability (external validity, applicability) of the trial findings

21-i) Generalizability to other populations

We have raised the issue of generalisability:

'On the other hand, this trial only included a specific target group of people presenting mild to moderate depressive symptoms, which affects generalizability to the larger population. In a naturalistic study it has been found that people seeking self-help interventions to improve well-being either show rather severe depression scores or else no signs of depression at all [41], whereas the present study targeted people with mild depressive symptoms. It would be insightful to examine if Psyfit is also effective in these other groups, in such a way that results could be generalized to a larger population.'

21-ii) Discuss if there were elements in the RCT that would be different in a routine application setting

At this moment, the application is how it was in the RCT setting.

22) CONSORT: Interpretation consistent with results, balancing benefits and harms, and considering other relevant evidence

22-i) Restate study questions and summarize the answers suggested by the data, starting with primary outcomes and process outcomes (use)

yes.
'Principal results
This randomized controlled trial examined the efficacy of an online self-help course aimed at promoting mental fitness and subsequent well-being. The results at 2-month follow-up show that in the intervention group participants made (marginally) significantly more improvement in the level of overall well-being and psychological well-being than participants in the waiting list control group. Furthermore, general health and vitality level were significantly enhanced, and depression and anxiety symptoms were reduced, in comparison to the waiting list control group. At 6-month follow-up, effects were maintained for depression and anxiety. All effects were in the small to medium range. Adherence analysis revealed no clear dose-response effect, although some larger effects appeared for people receiving at least a minimal part of the intervention. For well-being and depression, larger effects were found for higher educated people.

Comparison with previous work
The effects of taking part in Psyfit are comparable with effects of similar offline positive psychological interventions in self-help format with regard to well-being, but appear to be on average larger for depression [18,19]. When compared with studies of other online positive self-help interventions, the effect sizes in the current study are quite similar [29,30], and in some cases higher [27,28,33].'

22-ii) Highlight unanswered new questions, suggest future research

Yes:
'Regarding the future research agenda for OPPIs, emphasis should be placed on 1) increasing adherence and motivation by using persuasive design and/or providing minimal support, 2) examining mediation and moderation effects to determine what works best for whom, and 3) serving lower educated people better. The target group could be expanded to present more variety in the depressive symptom spectrum. This will likely help to strengthen the generalizability of these results to a larger group of people. Also, future studies of Psyfit or other OPPIs should consider the use of other control groups to overcome the limitations of a waiting list control group.'

Other information

23) CONSORT: Registration number and name of trial registry

Trial registration
The study is registered with the Netherlands Trial Register, part of the Dutch Cochrane Centre (NTR2126).'

24) CONSORT: Where the full trial protocol can be accessed, if available

Yes, at JMIR Res Protoc 2012;1(1):e2.

25) CONSORT: Sources of funding and other support (such as supply of drugs), role of funders

The study is funded by the Dutch Ministry of Health, Welfare and Sport.'

X26-i) Comment on ethics committee approval

The approval by the medical ethical commitee was stated:

'The study was approved by the Dutch Medical Ethics Committee for Mental Health Care, under registration number 9218.'

x26-ii) Outline informed consent procedures

We obtained online informed consent:

'and subsequently received an e-mail with information on the study and a link to the online consent form and baseline questionnaire.'

'....Adult participants (21 years and older) were included when informed consent was obtained '

X26-iii) Safety and security procedures

Privacy and safety of the intervention were secured.

People presenting a high level of depressive symptoms and/or suicidal thoughts/behavior at baseline were referred to the general practitionar or online national suicide platform.

There is a short statement in the article 'Those meeting any of these exclusion criteria were advised to seek professional help. ' but this was more extensivelely done in the protocol article.

X27-i) State the relation of the study team towards the system being evaluated

Conflicts of interest
Trimbos Institute is the developer of Psyfit.nl.'
